# Unlocking the potential of engineered exosomes for knee osteoarthritis therapy

**DOI:** 10.3389/fimmu.2026.1820504

**Published:** 2026-04-29

**Authors:** Hanyue Li, Yingfang Ao

**Affiliations:** 1 Tianjin Key Laboratory of Exercise Physiology and Sports Medicine, Institute of Sport, Exercise and Health, Tianjin University of Sport, Tianjin, China; 2 Department of Sports Medicine, Peking University Third Hospital, Institute of Sports Medicine of Peking University, Beijing, China; 3 Beijing Key Laboratory of Sports Injuries, Beijing, China; 4 Engineering Research Center of Sports Trauma Treatment Technology and Devices, Ministry of Education, Beijing, China

**Keywords:** biomaterials, cartilage repair, engineered exosomes, joint microenvironment, knee osteoarthritis, regenerative medicine, targeted therapy

## Abstract

Knee osteoarthritis (KOA) is a common age-related degenerative joint disease. Currently, there is a lack of effective treatments capable of altering its progression. Exosomes, as key mediators of intercellular communication, possess innate biocompatibility, low immunogenicity, and favorable barrier-penetrating capabilities, demonstrating potential in modulating the joint microenvironment. However, natural exosomes face challenges such as poor targeting specificity, limited drug-loading capacity, and a short half-life. To address these limitations, engineered exosomes have been developed through strategies including surface modification, drug-loading optimization, and integration with biomaterials, significantly enhancing their therapeutic efficacy in preclinical models. This review summarizes recent advances in the application of engineered exosomes for KOA treatment, with a focus on elucidating their molecular mechanisms in inhibiting inflammation, regulating chondrocyte function, maintaining extracellular matrix (ECM) homeostasis, modulating subchondral bone remodeling, and influencing pain pathways. Although preclinical studies have demonstrated promising therapeutic outcomes, the clinical translation of engineered exosomes still faces challenges, including standardized production, safety evaluation, optimization of targeting efficiency, and validation in large animal models. While Phase I safety data are available, the field currently lacks Phase II efficacy data or disease-modifying proof. Therefore, engineered exosomes represent a promising preclinical candidate requiring further validation through Phase II/III trials. Future research should focus on deepening mechanistic understanding, standardizing production processes, and conducting rigorous clinical trials to establish engineered exosomes as a viable therapeutic option for KOA.

## Graphical Abstract

**Figure:**
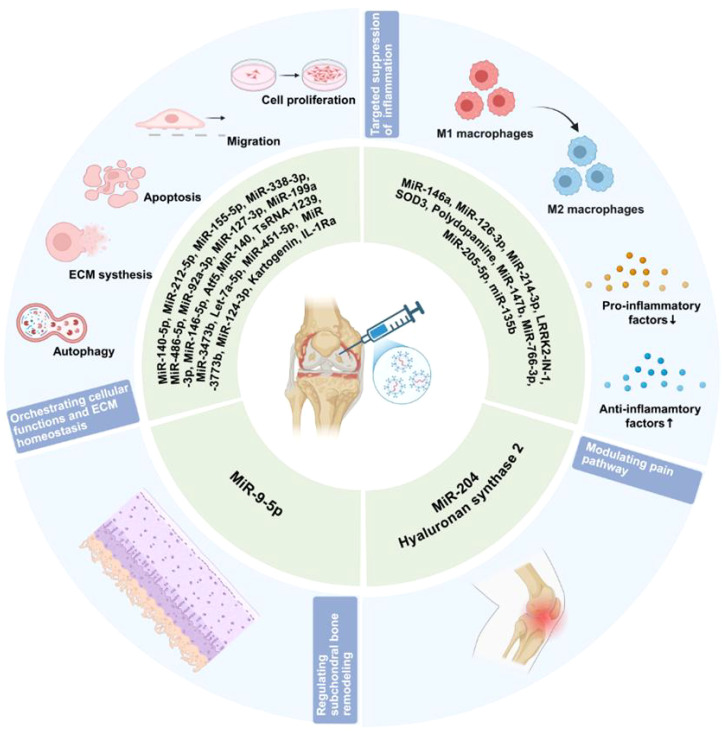
The proposed mechanisms and molecular cargo of engineered exosomes in KOA repair. This graphical abstract illustrates key biological processes influenced by engineered exosomes, including targeting suppression of inflammation, orchestrating cellular functions and ECM homeostasis, regulating subchondral bone remodeling, and modulating pain pathway. These functional effects are potentially mediated by various therapeutic cargoes, such as miRNAs (Figure was created with BioRender.com).

## 1 Introduction

Osteoarthritis (OA), the most prevalent form of arthritis in the aging population, is a chronic degenerative joint disease (1). It is characterized by the progressive structural deterioration of articular components, primarily cartilage, with concomitant involvement of adjacent bone, the synovial lining, and periarticular muscles (2). Recent epidemiological studies have underscored a persistent rise in the global prevalence of OA, which currently affects approximately 595 million people worldwide as of 2020, representing a 132.2% increase since 1990 (3). Notably, OA of the knee joint (KOA) accounts for nearly 85% of the global OA burden, making it the most commonly affected site (3). The management of KOA entails substantial economic ramifications, including significant direct medical costs—approximately double those of individuals without KOA—as well as indirect costs from work productivity impairment and loss of quality-adjusted life-years. In recognition of this mounting burden, the U.S. Food and Drug Administration (FDA) formally designated KOA as a “serious disease” in 2022, highlighting its profound clinical and public health significance.

Nevertheless, KOA persists as an intractable clinical challenge due to the absence of disease-modifying therapies (4). In clinical practice, reconstructive surgery, though improving quality of life in advanced cases, is confined to terminal-stage management, sometimes yielding incomplete pain resolution and carrying substantial reoperation risks. Nonsurgical interventions remain the therapeutic mainstay; however, pharmacological approaches (oral/topical analgesics) and physical modalities may provide merely palliative relief (5). Even adjunctive treatments such as intra-articular hyaluronic acid injections or glucocorticoid administration fail to arrest or reverse pathological progression (6). As such, driven by the pressing unmet clinical needs, intensive research endeavors have been increasingly directed toward developing novel therapeutics for the treatment of KOA.

It is widely acknowledged that effective repair of the damaged joints necessitates orchestrated bidirectional cellular crosstalk, primarily mediated through paracrine signaling pathways. Within this paradigm, extracellular vesicles, particularly exosomes, have emerged as key mediators of intercellular communication within the joint microenvironment (7). These naturally derived nanovesicles facilitate the transfer of bioactive molecules—such as nucleic acids, proteins, and lipids—between cells, thereby influencing processes central to KOA pathogenesis, including inflammation, matrix degradation, and cellular homeostasis (8). However, the clinical application of native exosomes is constrained by inherent limitations such as low targeting efficiency, rapid clearance, and variable cargo composition. To overcome these challenges, exosome engineering has arisen as a promising strategy to enhance their therapeutic potential in the preclinical models. Through genetic, chemical, and biomaterial-based modifications, engineered exosomes can be optimized for improved targeting, stability, and cargo loading (9, 10). This review comprehensively examines the molecular mechanisms by which engineered exosomes modulate the pathological processes of KOA and explores their therapeutic applications in preclinical models. By synthesizing recent advances and critically assessing ongoing challenges, this article aims to provide a foundational perspective for the continued development of exosome-based nanotherapeutics as a potential treatment modality for KOA.

## 2 Biogenesis, secretion, and cargo sorting of exosomes

**Figure 1:**
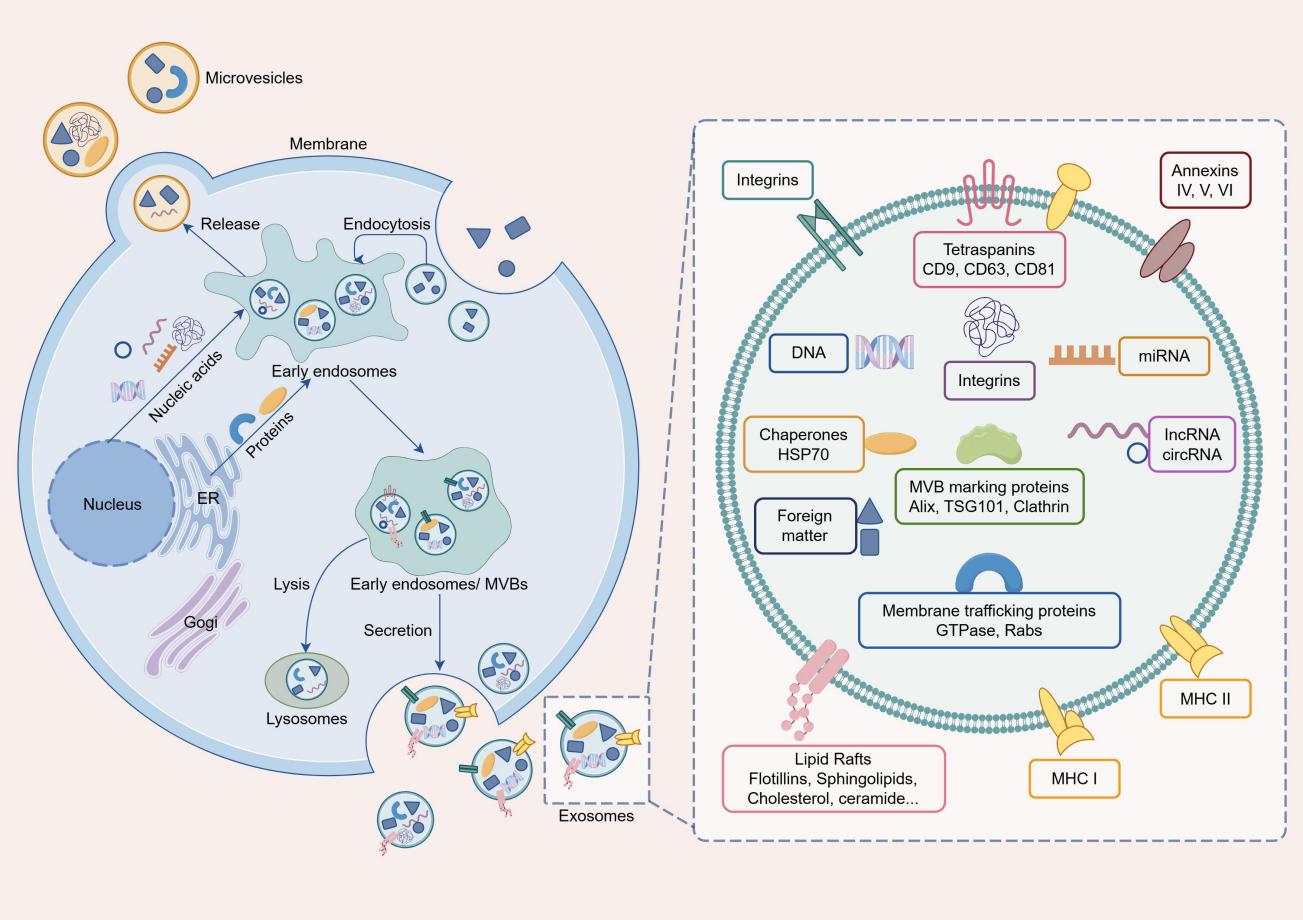
Biogenesis of exosomes. The process begins with the inward budding of the limiting membrane of an early endosome, leading to the formation of intraluminal vesicles (ILVs) within a multivesicular bodies (MVB). Some MVBs degrade into lysosomes and form endolysosomes for digestion within the intracellular space. When MVBs and the plasma membranes fuse, exosomes are released into the extracellular space. (Figure was created by Figdraw.).

Exosomes are nanoscale (30–150 nm) extracellular vesicles derived from the endosomal system, with their biogenesis tightly regulated by conserved molecular machinery (**Figure 1**) (11). Exosomes biogenesis is initiated by membrane invagination, forming vesicles coated with lattice proteins; these vesicles subsequently enter the cytoplasm to establish early endosomes (12). Within the Golgi complex and rough endoplasmic reticulum, differentially modified proteins and nucleic acids are selectively packaged to form intraluminal vesicles (ILVs) (13). Upon completion of late endocytosis, ILVs undergo further growth and maturation, ultimately forming late endosomes or multivesicular bodies (MVBs) (14). A portion of these MVBs follows the lysosomal degradation pathway, whereas the remainder fuse with the cell membrane and are ultimately excreted into the extracellular environment as exosomes (15).

Following their release into the extracellular space, exosomes traverse the degraded ECM and are subsequently internalized by recipient cells; however, the underlying mechanisms orchestrating this uptake process remain incompletely elucidated. Three principal uptake mechanisms have been proposed: (1) Ligands present on the exosomal membrane bind to surface receptors on target cells; (2) Exosomes directly with target cells membranes, releasing their internal cargo—including proteins and nucleic acids—to mediate intercellular information transfer; (3) Exosomes present surface-associated molecules that interact with receptors on target cells (16, 17). Despite the incomplete understanding of exosome uptake mechanisms, a key fact remains evident: exosomes mediate the transfer of biologically active molecules from donor cells to recipient cells. Compelling evidence supports the occurrence of this process across diverse cell types and disease contexts, including KOA (18). Once internalized by recipient cells, exosomes exert their functional effects through cargo-including nucleic acids (miRNAs, lncRNAs, circRNAs, mRNA), proteins (cytokines, growth factors, enzymes), and lipids (cholesterol, sphingolipids)—which are selectively sorted based on parental cell type and microenvironmental cues (14).

## 3 Engineering strategies for exosomes

Although extensive studies have reported that native exosomes possess promising therapeutic potential for KOA treatment (19), their clinical translation faces significant challenges. For example, native exosomes exhibit a limited capacity for loading exogenous therapeutic agents, and their natural cargo may lack optimal potency for treating severe or advanced KOA pathology (20).Upon administered, they are susceptible to rapid clearance by the mononuclear phagocyte system and renal filtration, which shortens their circulatory half-life and reduces their therapeutic time window (21). In contrast, engineered exosomes serve as a critical bridge between the biological basis of native exosomes and their translational potential in KOA treatment. Engineered exosomes are produced by modifying or manipulating native exosomes, which involves altering their cargo—such as proteins or nucleic acids—to enhance therapeutic properties, as well as modifying their surface characteristics to improve targeting and delivery to specific cells or tissues. In recent years, significant progress has been made in exosome engineering, with current methodologies primarily centered on two key approaches: pre-isolation and post-isolation modification.

### 3.1 Pre-isolation modification

Pre-isolation modification represents a primary engineering strategy wherein parent cells are genetically or biochemically modified to produce exosomes with desired properties. This approach typically involves introducing target molecules into donor cells through methods such as direct transfection, as well as chemical or physical treatment of parent cells. Following the cellular uptake or expression of these molecules, the endosomal pathway facilitates their packaging into the ILVs of multivesicular bodies MVBs, which are subsequently secreted as exosomes carrying the therapeutic cargo (22). A prominent strategy for engineering exosomes involves the direct treatment of parent cells with chemical or physical stimuli. This approach is predicated on the central premise that since exosomes originate from parent cells, their characteristics directly reflect the physiological and biochemical alterations induced in these cells. Chemically engineered exosomes for KOA repair are commonly produced by incubating donor cells with various agents, including inflammatory cytokines such as TNF-α and IL-1β, hormones like parathyroid hormone (PTH), growth factors including TGF-β1, and numerous natural bioactive plant extracts. Following this therapeutic incubation, the donor cells acquire new biological properties, which are subsequently transferred to the exosomes they release upon appropriate stimulation. These engineered exosomes are then isolated and purified using techniques such as ultracentrifugation, polymer-based precipitation, immunoaffinity capture, or microfluidics (23). Similarly, physical modification of parent cells presents another viable approach. For instance, given that oxygen concentration is crucial for cellular homeostasis, Shen et al. demonstrated that hypoxia preconditioning can endow exosomes with an enhanced capacity for cartilage regeneration (24). Furthermore, as mechanical force is a key regulator in KOA progression, studies have shown that chondrocytes subjected to mechanical stress secrete exosomes enriched with miR-9-5p, which confers a protective effect against KOA pathogenesis (25). These methods do not require specialized equipment, while the physical and functional features of exosomes are well maintained. Alternatively, selected parental cells can be genetically modified using viral or non-viral vectors to load therapeutic proteins or oligonucleotides, leading to increased or silenced gene expression. It is also possible to transfect parent cells with the desired gene to produce exosomes loaded with therapeutic molecules (26). This process can be accomplished via chemical methods, electroporation, or viral vector-mediated delivery. The general procedure involves collecting donor cells, introducing the desired cargo via transfection, harvesting exosomes from the culture supernatant, and removing cellular debris and large impurities to obtain purified, drug-loaded exosomes. In practice, the application of the transfection methods requires careful consideration of multiple factors, including the type of target cells, the physicochemical properties of the cargo (e.g., size, charge, hydrophilicity), and the intended therapeutic outcome. Additionally, maintaining cell viability and exosome yield during transfection is critical. It is important to emphasize that the preparation of drug-loaded exosomes demands rigorous experimental design and validation to identify the most suitable cargo molecules and optimal processing conditions.

### 3.2 Post-isolation modification

Post-isolation modification encompasses range of techniques applied to exosomes after purification, allowing precise engineering of their surface properties and internal cargo to improve therapeutic efficacy and targeting specificity. This approach is particularly valuable when pre-isolation strategies are insufficient for incorporating specific functional elements, or when working with sensitive cargo requiring strict loading control. Surface modification strategies generally include chemical and genetic approaches (27). Genetic engineering is a widely adopted method for modifying exosome surfaces. It typically involves constructing a fusion plasmid that links a gene encoding a target protein to that of an exosome scaffolding protein (28). The resulting exosomes often display functionalized surfaces featuring targeting peptides, sequences, or antibodies, enabling targeted delivery to specific cells or tissues. Commonly used scaffold proteins include type I transmembrane proteins (e.g., Lamp2), tetraspanins, and peripheral membrane proteins, among which Lamp2b is the most extensively applied due to its N-terminal extracellular domain that supports effective fusion with targeting moieties (29). For instance, Liang et al. generated chondrocyte-targeted exosomes by fusing a chondrocytes-affinity peptide (CAP) to Lamp2b (30). Their findings demonstrated that CAP-modified exosomes exhibited enhanced targeting to cartilage tissue, supporting their potential as stable drug delivery vehicles for cartilage-specific applications (30). In addition to genetic engineering, chemical modification, encompassing both covalent and non-covalent strategies, serves as another prominent technique for functionalizing exosomal surfaces. Covalent approaches, such as click chemistry or amine-reactive crosslinking, allow the stable conjugation of targeting ligands to the exosomal membrane, thereby enhancing targeting specificity (31). Non-covalent strategies offer versatile alternatives, including hydrophobic insertion—where lipid-anchored ligands spontaneously incorporate into the membrane—and electrostatic adsorption, which utilizes the attraction between positively charged modifiers and the negatively charged exosomal surface (32). On the other hand, cargo loading constitutes another essential dimension of post-isolation modification. It involves loading drugs onto the surface or into the interior of purified exosomes using physical or chemical methods. Common techniques include electroporation, sonication, freeze-thawing, saponification, extrusion, co-incubation, and transfection (33). Moreover, biomaterial-assisted exosomes also fall within the scope of post-isolation modification for producing engineered exosomes. Biomaterials such as scaffolds and hydrogels can enhance pharmaceutical acceptability by prolonging storage stability and modulating release kinetics (34). Despite its versatility, post-isolation modification presents challenges including particle aggregation, structural damage, and batch-to-batch variability. These issues necessitate careful optimization of reaction parameters such as pH, temperature, and reagent ratios. Comprehensive characterization using nanoparticle tracking analysis, western blotting, and electron microscopy is essential to confirm successful modification and assess vesicle integrity. When properly optimized, this approach substantially broadens the therapeutic potential of exosomes, enabling the design of advanced drug delivery systems with tailored targeting and controlled release profiles for applications in precision medicine.

## 4 Core pathogenesis of KOA: target for intervention

**Figure 2:**
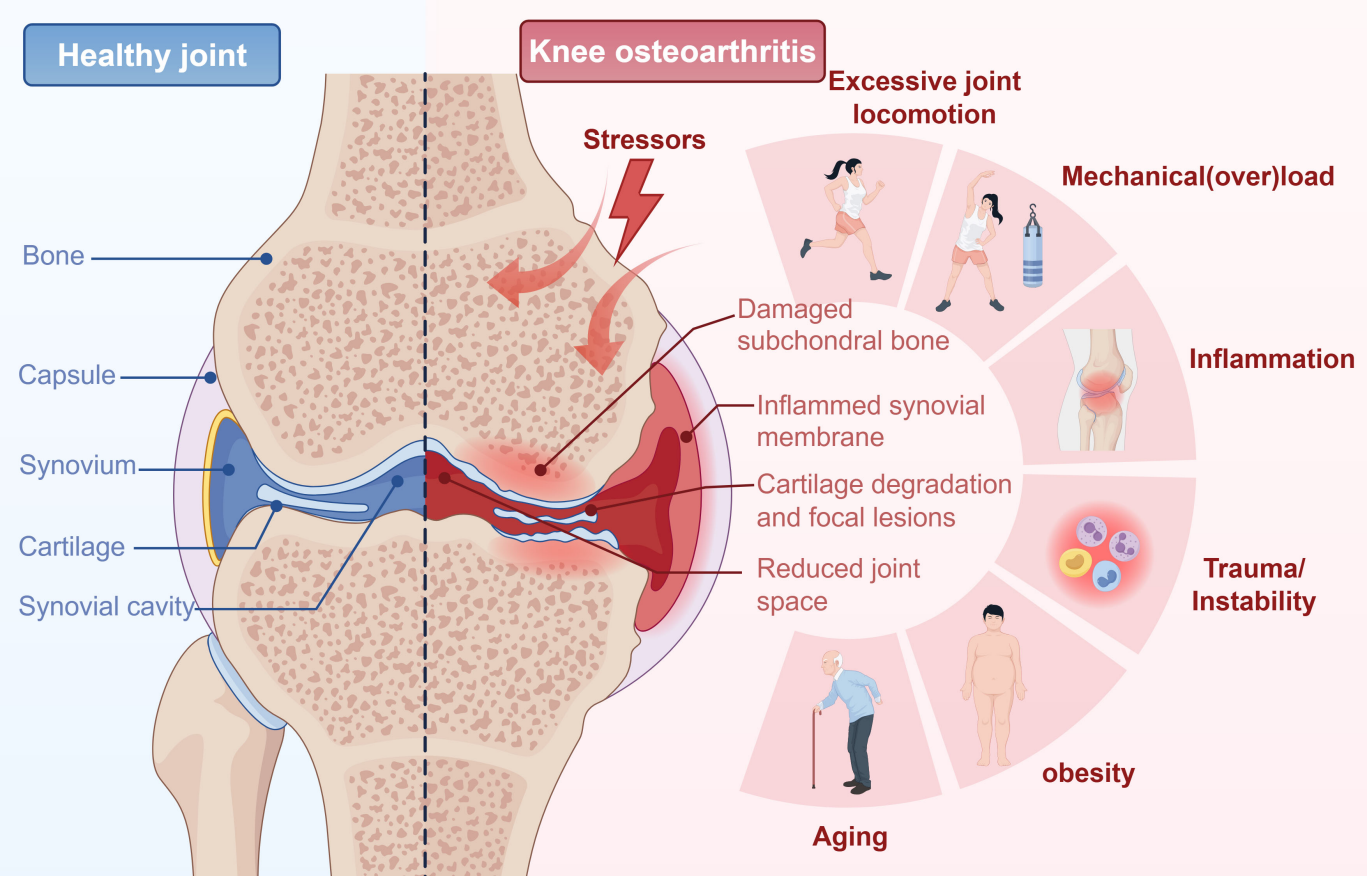
Schematic diagram illustrating the pathogenesis of knee osteoarthritis (KOA). The diagram contrasts a healthy joint (left) with an osteoarthritic joint (right). KOA development is initiated by various stressors, including mechanical overload from excessive joint locomotion, inflammation, trauma/instability, obesity, and ageing. These factors drive a pathological cascade characterized by key structural changes: damaged subchondral bone, inflamed synovial membrane, cartilage degradation with focal lesions, and reduced joint space. (Figure was created by Figdraw.).

KOA is a degenerative joint disorder characterized by a complex, multifactorial pathophysiology involving multiple articular tissues and dysregulated biological processes (35). A central paradigm in the pathogenesis of KOA is that joint homeostasis is disrupted by a combination of biomechanical stressors and systemic risk factors. Key initiating factors include mechanical overload from excessive joint loading or malalignment, inflammation, obesity, ageing, and joint instability secondary to trauma. Collectively, these stressors trigger a cascade of pathological changes in the synovium, subchondral bone, and cartilage, which together drive disease development and progression (**Figure 2**). Central to KOA pathogenesis is the progressive degradation of articular cartilage. Cartilage serves as a critical cushioning tissue within the joint; however, under conditions of aging, abnormal mechanical stress, or metabolic dysregulation, chondrocytes undergo dysfunctional activity (36). This dysfunction further suppresses matrix synthesis while concurrently stimulating the activity of matrix-degradation enzymes, leading to progressive joint destruction and aberrant remodeling (37). Key enzymes implicated in matrix degradation in osteoarthritic joints include aggrecanase and collagenases, both of which are members of the matrix metalloproteinase (MMP) family, as well as various serine and cysteine proteases. Matrix degradation in early KOA may be attributed to MMP-3 and aggrecanase, particularly the a disintegrin and metalloproteinase with thrombospondin motifs 5 (ADAMTS-5). Progressive loss of the ECM in osteoarthritic cartilage gradually impairs its capacity to absorb mechanical stress, resulting in excessive load transfer to the subchondral bone (38). This further disrupts the delicate balance between osteoclast-mediated bone resorption and osteoblast-mediated bone formation, leading to heterogeneity in subchondral density and stiffness. Such heterogeneity generates localized shear forces that further exacerbate cartilage deformation and damage (39). Moreover, synovial inflammation is also regarded as a key pathological feature contributing to the progression of KOA. This inflammation is primarily driven by the presence of neutrophils and the prevalence of proinflammatory M1 macrophages, alongside the scarcity or absence of anti-inflammatory M2 macrophages. This macrophage phenotypic imbalance not only initiates the inflammatory response but also sustains it within the synovial microenvironment (40). Moreover, neutrophils, a major subset of infiltrating immune cells in the inflamed synovium, mediate synovial and peri-articular tissue damage through the massive release of reactive oxygen species (ROS) and MMPs. This tissue damage, in turn, triggers a positive feedback loop: the release of damage-associated molecular patterns (DAMPs) from injured cells promotes further secretion of proinflammatory factors, ultimately accelerating the degradation of the articular cartilage ECM (41). In addition to these immune-mediated processes, emerging evidence highlights a pivotal role of neuroimmune crosstalk in sustaining KOA-associated synovial inflammation. Sensory nerve fibers in the synovium release neuropeptides (e.g., substance P, calcitonin gene-related peptide) upon activation, which not only amplify pain signaling but also modulate immune cell activity (e.g., enhancing macrophage cytokine production and neutrophil chemotaxis), thereby reinforcing the inflammatory microenvironment (42). Despite ongoing efforts to unravel its complexities, KOA pathogenesis remains incompletely understood. However, this interconnectedness explains the limited efficacy of single-target therapies, underscoring the need for interventions that address multiple pathological nodes.

## 5 Molecular mechanisms and therapeutic opportunities of engineered exosomes

Through the application of various engineering strategies, exosomes have been empowered with enhanced targeting capability, efficient loading of therapeutic molecules, and improved stability, making them highly suitable for the treatment of KOA. To systematically elucidate their functions, both *in vitro* and *in vivo* studies have been conducted. *In vitro* investigations have demonstrated that engineered exosomes modulate pivotal cellular processes implicated in KOA pathogenesis via various signaling pathways, including inhibiting inflammation, orchestrating cellular functions and ECM homeostasis, managing subchondral bone remodeling, and modulating pain pathway. Moreover, *in vivo* studies have robustly validated the translational potential of these mechanisms, confirming that engineered exosomes elicit tangible therapeutic benefits in animal models of KOA (**Supplementary Table 1**).

### 5.1 Targeted suppression of inflammation

The pathological progression of KOA is largely driven by sustained, low-grade inflammation. An initial mechanical injury or other pathological stimuli activate the immune system, triggering the release of proinflammatory cytokines like IL-1β, TNF-α, and IL-6, which initiates an inflammatory cascade (43). This response in turn promotes the recruitment of immune cells such as macrophages, polarizing them toward a pro-inflammatory M1 phenotype and further amplifying the secretion of inflammatory mediators. Concurrently, inflammation-associated oxidative stress increases intracellular ROS and aggravates the loss of chondrocyte phenotypic stability and the ECM degradation, causally creating a microenvironment favoring cartilage lesions. Therefore, therapeutic strategies to inhibit inflammatory signaling cascades and reduce ROS level have been recognized as promising approach for treating KOA.

#### 5.1.1 Targeting core inflammatory pathways

A primary focus has been the inhibition of pivotal signaling axes such as TLR4/TRAF6/NF-κB, whose activation promotes catabolic and pro-inflammatory gene expression (44). In this regard, activation of TLR4 by damage-associated molecular patterns (DAMPs) initiates a signaling cascade involving TRAF6, leading to NF-κB nuclear translocation (45). This, in turn, promotes the transcription of proinflammatory cytokines (e.g., IL-6, TNF-α) and matrix-degrading enzymes (e.g., MMP-1, MMP-3, MMP-13), contributing to inflammation amplification and ECM destruction (46). Engineered exosomes offer a targeted approach to interrupt this pathway at multiple points.

A common strategy involves enriching exosomes with specific anti-inflammatory miRNAs. For instance, fibroblast-like synoviocytes (FLS)-derived exosomes overexpressing miR-146a attenuate TLR4/TRAF6/NF-κB signaling and suppress pro-inflammatory factor expression, thereby improving cartilage repair and alleviating KOA progression (47). Similarly, exosomes from miRNA-modified rat FLSs (e.g., miR-126-3p and miR-214-3p) significantly suppress IL-1β, IL-6, and TNF-α expression, protecting chondrocytes under inflammatory conditions (48, 49).

#### 5.1.2 Orchestrating macrophage polarization: a central hub for inflammation resolution

**Figure 3:**
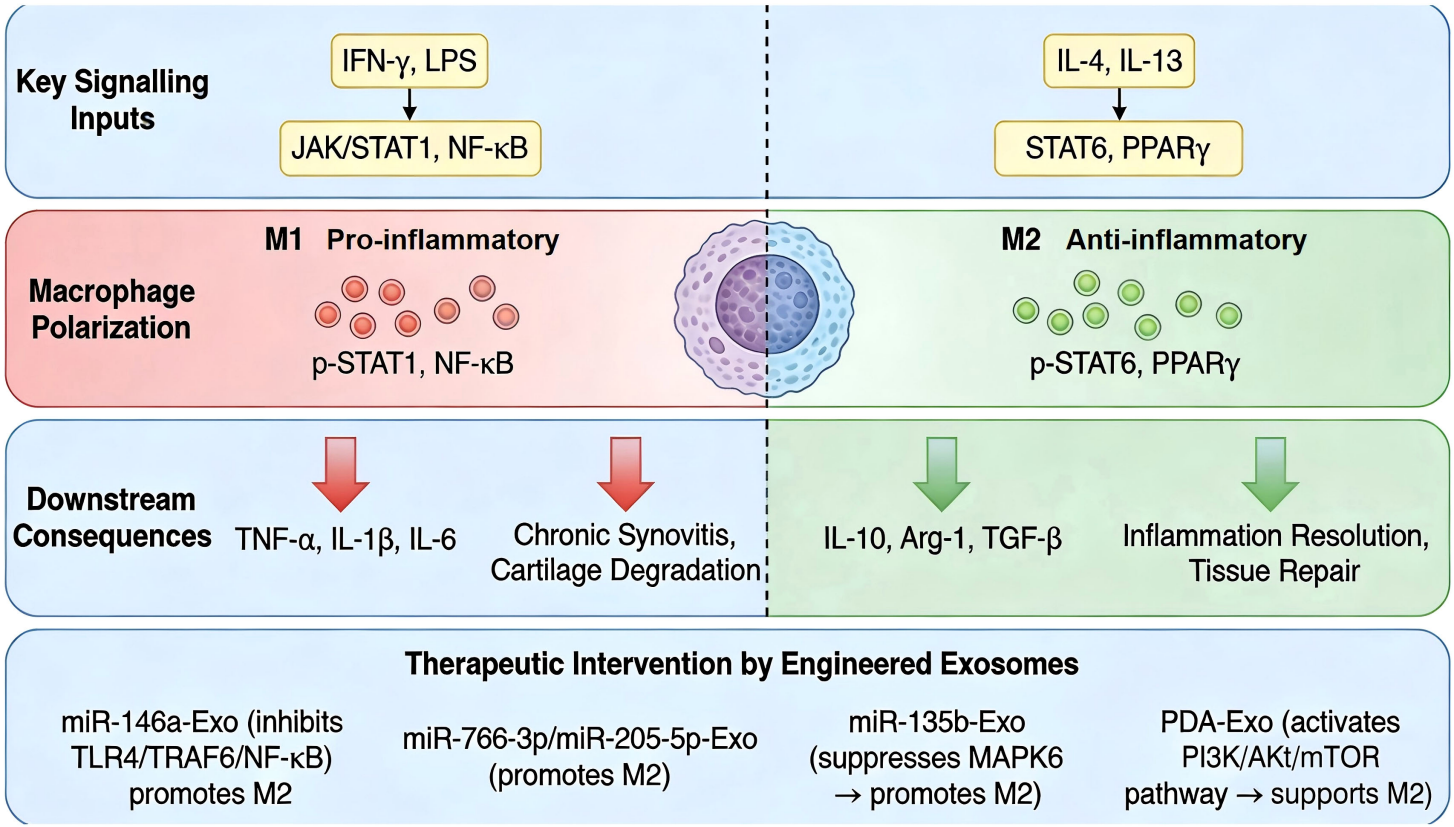
Schematic diagram summarizing macrophage polarization, key signaling inputs, downstream pathological consequences, and therapeutic intervention by engineered exosomes in knee osteoarthritis (KOA). Key signaling inputs driving macrophage polarization: M1 polarization is induced by IFN-γ and LPS via JAK/STAT1 and NF-κB pathways; M2 polarization is induced by IL-4 and IL-13 via STAT6 and PPARγ pathways. Macrophage polarization states: M1 (pro-inflammatory) macrophages are characterized by p-STAT1 and NF-κB activation; M2 (anti-inflammatory) macrophages are characterized by p-STAT6 and PPARγ activation. Downstream pathological consequences: M1 macrophages produce pro-inflammatory cytokines (TNF-α, IL-1β, IL-6), contributing to chronic synovitis and cartilage degradation in KOA. Therapeutic intervention by engineered exosomes: Exosomes derived from various sources deliver bioactive cargo (e.g., miR-146a, miR-766-3p, miR-205-5p) to modulate macrophage polarization. miR-146a-overexpressing exosomes inhibit TLR4/TRAF6/NF-κB signaling, reducing M1 polarization and promoting M2 shift; exosomes enriched with miR-766-3p and miR-205-5p directly promote M2 polarization, rebalancing the cytokine milieu and facilitating inflammation resolution and cartilage repair. This schematic integrates the molecular drivers, cellular phenotypes, and therapeutic strategies targeting macrophage polarization in KOA. Figure was manually drawn by the authors using Microsoft PowerPoint.

The imbalance between pro-inflammatory M1 and anti-inflammatory M2 macrophages is a critical driver of chronic synovitis in KOA. Its polarization state is governed by distinct signaling networks (50). Classical M1 polarization is primarily driven by factors such as IFN-γ and LPS, which signal through JAK/STAT1 and NF-κB pathways, leading to the production of pro-inflammatory cytokines (TNF-α, IL-1β, IL-6) (51). Conversely, alternative M2 activation is commonly induced by IL-4 and IL-13, engaging STAT6 and PPARγ pathways to promote the expression of anti-inflammatory mediators like IL-10, Arg-1, and TGF-β (52). Engineered exosomes re−establish immune homeostasis by shifting the balance toward a reparative M2 phenotype. Mechanistically, exosomal miR−146a reduces M1 polarization through NF-κB (47); exosomes from IL-1β-primed MSCs (enriched in miR-766-3p and miR-205-5p) directly promote M2 polarization (53); and TGF-β1-primed MSC exosomes deliver miR-135b to suppress MAPK6 signaling, facilitating chronic inflammation resolution (54). Beyond miRNAs, polydopamine-incorporated exosomes activate PI3K/Akt/mTOR to support M2 polarization (55). Even native chondrocyte-derived exosomes can reprogram macrophage phenotype, highlighting complex joint crosstalk (56). Collectively, these examples illustrate that engineered exosomes can effectively tip the balance from a destructive M1-dominant state to a reparative M2-dominant state by modulating various signaling cascades and transcription factor activity (**Figure 3**).

#### 5.1.3 Delivery of anti-inflammatory and antioxidant cargos

Exosomes also serve as vehicles for small-molecule inhibitors and antioxidant enzymes. Cartilage-targeted exosomes (WYRGRL peptide) delivering LRRK2-IN-1 block IL-1β-induced NF-κB activation (57); puerarin-loaded exosomes inhibit IκBα degradation and p65 nuclear translocation (58). On the other hand, oxidative stress, particularly ROS overproduction, contributes to chronic inflammatory damage and forms a mutual amplification loop with inflammation (59). Using single-cell sequencing, Cao et al. identified superoxide dismutase 3 (SOD3) as a key antioxidant and constructed SOD3-loaded exosomes that sustain ROS neutralization, prevent ECM degradation and reduce inflammatory infiltration (60). Thus, exosomes engineered with targeting ligands and loaded with anti-inflammatory or antioxidant agents enable precise modulation of inflammation and oxidative stress in KOA.

### 5.2 Orchestrating cellular functions and ECM homeostasis

Beyond modulating inflammation, engineered exosomes play a pivotal role in directly promoting a pro-regenerative microenvironment within osteoarthritic joints by coordinately regulating key cellular processes—including cell migration, proliferation, apoptosis, and ECM homeostasis. A crucial and complementary mechanism involves the restoration of cellular homeostasis through the regulation of autophagy, essential processes for the removal of damaged components in the quiescent chondrocytes of articular cartilage.

#### 5.2.1 miRNA-mediated regulation of key signaling pathways

Several studies have engineered exosomes to carry miRNAs that target Runx2, a key regulator of chondrocyte hypertrophy. For example, exosomal miR-155-5p, miR-338-3p or miR-486-5p consistently stimulate chondrocyte proliferation, suppress apoptosis and enhance ECM synthesis (61–64). However, the role of Runx2 appears complex, as complete silencing may exacerbate disease progression (65), warranting further investigation into its therapeutic window. Other effective strategies include exosomal miR-140-5p targeting VEGFA (66), miR-212-5p suppressing ELF3 phosphorylation (63), and miR-92a-3p or miR-127-3p inhibiting Wnt/β-catenin signaling (67, 68). Chondrocyte-derived exosomes overexpressing miR-95-3p target HDAC2/8 3’-UTRs, maintaining ECM homeostasis and preventing cartilage degradation *in vitro (*69). Collectively, these studies demonstrate that miRNA-enriched exosomes can modulate chondrocyte function through diverse signaling pathways, though direct comparisons of their relative efficacy are lacking.

#### 5.2.2 Surface engineering for targeted delivery

**Figure 4:**
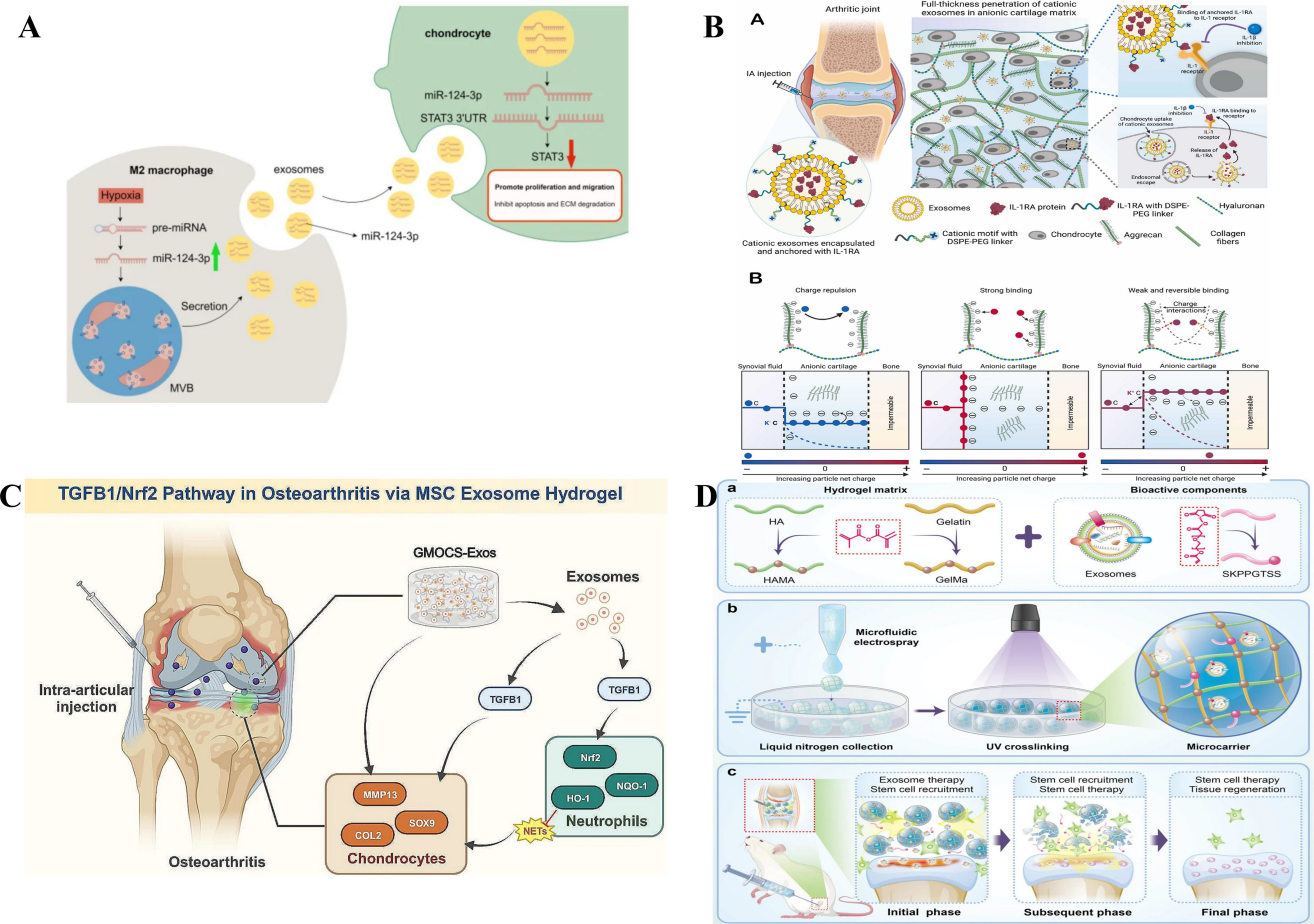
Therapeutic applications of engineered exosomes in knee osteoarthritis (KOA). **(A)** Charge-mediated targeted drug delivery to cartilage. **(a)** Schematic depiction of cationic exosomes loaded with IL-1RA permeating cartilage tissue via electrostatic attraction; **(b)** Negatively charged carriers experience electrostatic repulsion from glycosaminoglycans (GAGs) in cartilage, leading to a reduced concentration gradient at the synovial fluid–cartilage interface (from C to K−C) and consequently lower intra-tissue diffusion rates. Adapted from Ref (71).. under a CC BY license. **(B)** Hypoxic M2 macrophage–derived exosomes enriched with miR-124-3p promote chondrocyte proliferation and migration while inhibiting apoptosis and ECM degradation via STAT3 Targeting. Adapted from Ref (72). under a CC BY license. **(C)** Exosomes derived from bone marrow mesenchymal stem cells (BMSCs) encapsulated in extracellular matrix (ECM)–mimicking hydrogels modulate the TGFB1/Nrf2 Signaling Pathway. Adapted from Ref (73). under a CC BY license. **(D)** Schematic illustration of exosome-encapsulated stem cell recruitment microparticles for KOA Therapy. **(a)** Composition of the hydrogel matrix and bioactive components within the microcarriers. **(b)** Fabrication process of the functionalized microcarriers. **(c)** Application of the microcarriers in the treatment of KOA. Adapted from Ref (74). under a CC BY license.

Surface modifications can improve exosome retention, targeting and efficacy. CAP-peptide-conjugated exosomes deliver miR-140 or siRNA against MMP13 to injury sites, protecting articular cartilage (30, 70). A cationic peptide carrier (CPC) enables full-thickness cartilage penetration and weeks-long retention of IL-1RA, outperforming unmodified therapeutics (**Figure 4A**) (71). Moreover, dendritic cell-derived exosomes displaying E7-Lamp2b target synovial MSCs to deliver kartogenin(KGN), promoting functional cartilage repair (75). These advances highlight the potential of surface engineering, but further optimization for different cell types and patient populations is needed.

#### 5.2.3 Induction of general autophagy for cellular homeostasis

Autophagy, an evolutionarily conserved mechanism for eliminating defective organelles and macromolecules, is essential for maintaining cellular homeostasis, particularly in postmitotic tissues like cartilage (76–78). Engineered exosomes have been shown to alleviate KOA by inducing autophagy, often through inhibiting the negative regulator mTOR (79, 80). CAP-modified exosomes delivering miR-199a-3p regulate mTOR to promote autophagic flux (81). Fucoidan-pretreated MSC exosomes enhance autophagy by targeting TRAF6 and inhibiting the PI3K/AKT/mTOR axis, with miR-146-5p playing a key role (82). Advanced approaches using modRNA encoding Atf5 promote chondrocyte autophagy via mTOR/Ulk1 inhibition, linked to mitochondrial unfolded protein response activation (83). In summary, engineered exosomes can affect the level of autophagy by targeting downstream autophagy-related regulatory pathways, such as mTOR or TRAF6, thereby promoting KOA repair.

#### 5.2.4 Activation of mitophagy for mitochondrial homeostasis

Mitochondrial dysfunction is a hallmark of KOA pathogenesis, and mitophagy (selective mitochondrial autophagy) is critical for eliminating damaged organelles. The PINK1-Parkin pathway is the best-characterized mitophagy mechanism (84). In healthy mitochondria, PINK1 is continuously imported and cleaved; upon depolarization, PINK1 stabilizes on the outer membrane, recruiting and activating Parkin, which ubiquitinates outer membrane proteins, targeting mitochondria for autophagic degradation (85). However, in KOA, PINK1 expression is downregulated in osteoarthritic chondrocytes, compromising Parkin recruitment. Oxidative stress can inactivate Parkin via S-nitrosylation (86). The resulting accumulation of dysfunctional mitochondria drives a vicious cycle: excessive ROS production causes oxidative damage, activates NF-κB inflammatory signaling, and triggers cytochrome c release, promoting chondrocyte apoptosis and ECM degradation (87, 88). This mitophagic failure represents a critical nexus linking mitochondrial health, inflammation, and cell death. Engineered exosomes offer a targeted means to restore mitophagy by delivering therapeutic cargo that intervenes at specific regulatory nodes. For example, exosomal miR-140 from urine-derived stem cells downregulates calpain 1 (CAPN1), a protease that inactivates mitophagy proteins, improving mitochondrial morphology and reducing ROS (89). Similarly, exosome-encapsulated tsRNA-12391 binds ATAD3A (a negative regulator of PINK1), preventing PINK1 degradation and enhancing mitophagic flux (90). Exosomes delivering GRPEL1 (a positive regulator of PINK1) activate PINK1-mediated mitophagy and promote cartilage repair (91). Furthermore, strategies targeting the PINK1/Parkin pathway through gene editing (92) or the transfer of functional mitochondrial components via mitochondrial extracellular vesicles (MitoEVs) represent promising future directions for restoring mitophagic capacity in osteoarthritic chondrocytes (93). Together, these studies demonstrate that engineered exosomes can restore mitophagy in osteoarthritic chondrocytes by targeting key regulatory nodes such as CAPN1, ATAD3A, or GRPEL1, thereby reactivating the PINK1-Parkin pathway. These advances pave the way for clinically translatable exosome therapies that address the underlying mitochondrial dysfunction in KOA.

### 5.3 Regulating subchondral bone remodeling

KOA involves biphasic subchondral bone changes: early resorption (decreased plate thickness, increased vascularization) followed by late sclerosis (increased density, osteophyte formation) (94). This temporal heterogeneity demands therapeutics that dynamically regulate osteoclast and osteoblast activity. Engineered exosomes have shown promise. TGF-β1-modified BMSC exosomes suppress RANKL-induced MAPK and Smad signaling to attenuate osteoclastogenesis, inhibit pathological H-type vessel formation via PDGF-BB downregulation, and reduce pain mediators (netrin-1, CGRP) (95). Separately, mechanically stressed chondrocytes release exosomes enriched in miR-9-5p (via circStrn3 downregulation), which targets KLF5 in osteoblasts to suppress osteogenic differentiation, alleviating cartilage degradation and bone remodeling (25). Evidence from related fields (osteoporosis, periodontitis) suggests that platelet-derived exosomal miRNAs (miR-21, miR-223, miR-214, miR-155) modulate RANKL/OPG balance, and M2 macrophage-derived exosomal miR-1227-5p inhibits osteoclastogenesis via OSCAR targeting (96, 97). While these studies originate from osteoporosis and periodontitis research, they identify mechanistic pathways—particularly RANKL/OPG signaling and miRNA-mediated osteoclast regulation—that may be translatable to KOA subchondral bone remodeling. These pathways may be translatable to KOA. Despite these advances, studies specifically investigating engineered exosomes for regulating subchondral bone remodeling during KOA development remain limited (**Supplementary Table 2**). The field would benefit from systematic investigation of exosome-mediated RANKL/RANK/OPG axis modulation in the context of KOA’s biphasic bone pathology, as well as exploration of exosome-based strategies to differentially target early-stage bone resorption versus late-stage sclerosis. This represents an important direction for future KOA research.

### 5.4 Modulating pain pathway

**Figure 5:**
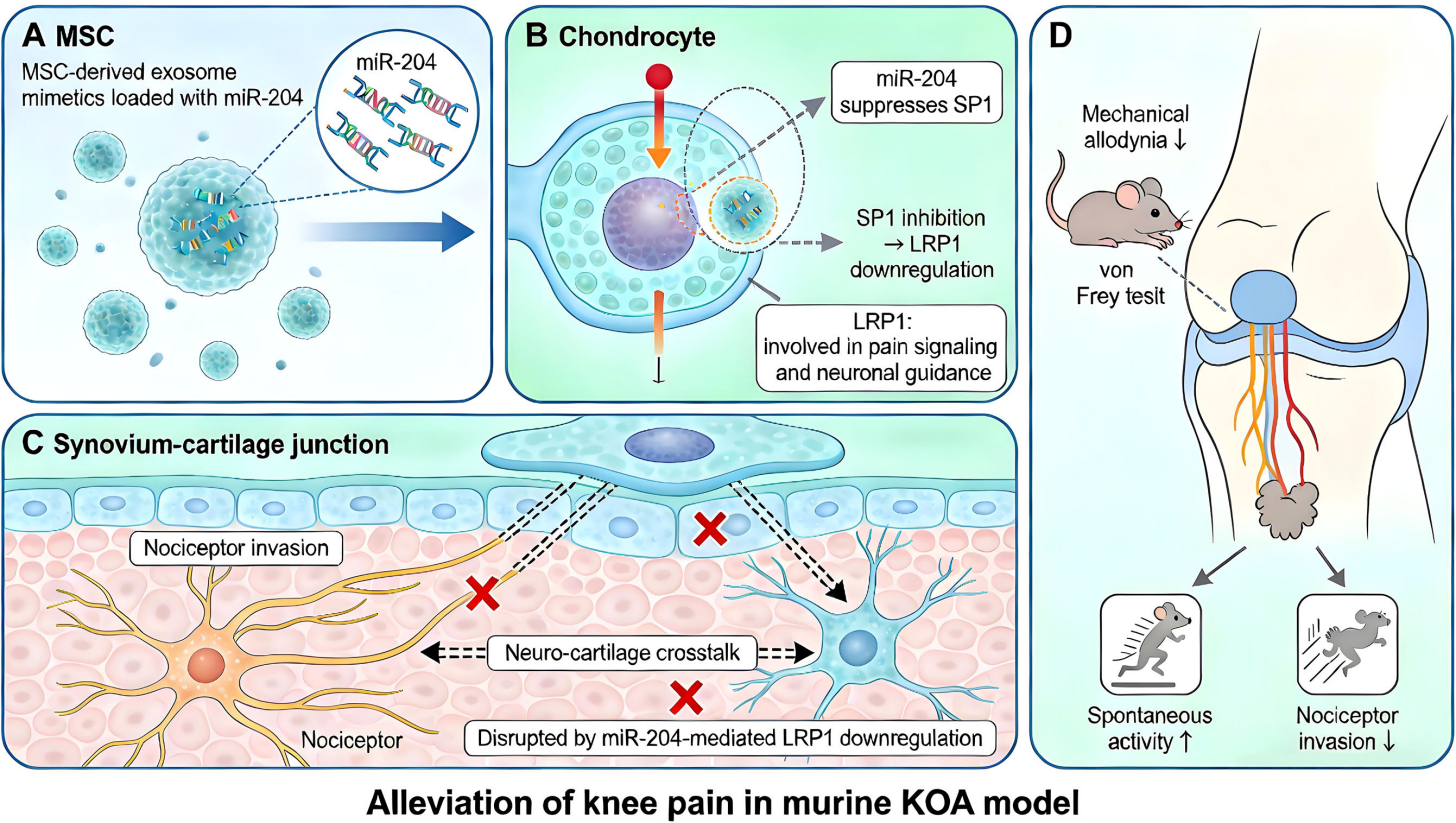
Schematic illustration of the analgesic mechanism of miR-204-loaded exosome mimetics in knee osteoarthritis (KOA). **(A)** Exosome mimetics derived from mesenchymal stromal cells (MSCs) are engineered to carry miR-204 and are delivered to chondrocytes. **(B)** Following intracellular delivery, miR-204 suppresses the transcription factor SP1, leading to downregulation of LDL receptor-related protein 1 (LRP1)—a molecule involved in pain signaling and neuronal guidance. **(C)** Downregulation of LRP1 disrupts neuro-cartilage crosstalk at the synovium−cartilage junction and inhibits the invasion of nociceptor nerve fibers into this region. **(D)** These molecular and cellular events collectively result in the attenuation of pain-related behaviors, including reduced mechanical allodynia and increased spontaneous activity, in a murine model of KOA. Figure was manually drawn by the authors using Microsoft PowerPoint.

Engineered exosomes mitigate KOA pain through multiple mechanisms. Lu et al. showed that MSC-derived exosome mimetics delivering miR-204 into chondrocytes attenuated pain-related behaviors by inhibiting nociceptor invasion at the synovium-cartilage junction. Mechanistically, exosomal miR-204 suppresses SP1, leading to downregulation of LRP1, thereby disrupting neuro-cartilage crosstalk (**Figure 5**) (98). Targeting neurogenic inflammation, exosomes from CGRP-antagonist-transduced infrapatellar fat pad MSCs express neprilysin (CD10) to degrade Substance P, reduce neuronal pro-inflammatory markers, and promote anabolic gene expression in chondrocytes, attenuating pain behaviors and preserving cartilage (99). An innovative pH-sensitive exosome platform loaded with hyaluronan synthase 2 (HAS2) selectively fuses with chondrocyte membranes under acidic conditions, enabling sustained high-molecular-weight hyaluronan production. A single injection provides analgesic effects for >4 weeks, outperforming conventional HA therapy, while also reducing pro-inflammatory cytokines and synovial macrophage infiltration (100). Thus, engineered exosomes can directly target neuronal signaling pathways (SP, CGRP), modulate chondrocyte-neuron communication (miR-204/SP1/LRP1), or reprogram chondrocytes to restore a protective microenvironment (HAS2 delivery) (**Supplementary Table 2**). Further studies are needed to explore additional pain pathways, including neuroimmune interactions and central pain mechanisms.

### 5.5 Enhancing therapeutic potency through cellular preconditioning

Preconditioning parental cells with specific stimuli generates exosomes with enhanced, built−in therapeutic cargo without direct engineering. For immunomodulation, TNF-α or IL-1β preconditioning yields exosomes that suppress NF−κB (e.g., via miR−147b) (101, 102). For cartilage repair, preconditioning with PTH (enriching miR-3473b), tropoelastin (enriching miR-451-5p), or decellularized ECM (enriching miR-377-3p) promotes chondrocyte migration, proliferation and matrix synthesis while inhibiting degradation (103–106). TGF−β1 preconditioning enriches miR−135b, which negatively regulates Sp1 to promote proliferation (107). Natural compounds also show promise: curcumin-stimulated MSC exosomes (miR-143, miR-124) inhibit ROCK1/TLR9/NF-κB signaling (108); cinnamaldehyde-pretreated BMSC exosomes suppress ECM degradation, potentially via NF−κB and MAPK (109). Hypoxic preconditioning is another potent strategy. Exosomes from hypoxic ADSCs are enriched in cartilage-related miRNAs (miR-381-3p, miR-122-5p, etc.) that promote ECM synthesis and mitigate inflammation (110). Hypoxia-pretreated M2 macrophage−derived exosomes carrying miR-124-3p target STAT3 activation to promote chondrocyte proliferation and inhibit apoptosis (**Figure 4B**) (72). Cellular preconditioning thus provides a versatile, efficient method to generate potent exosomes for KOA repair while avoiding direct engineering complexities. However, the molecular basis of cargo enrichment during preconditioning remains incompletely understood and warrants further investigation.

### 5.6 Synergistic combinations with biomaterial systems for enhanced delivery

**Figure 6:**
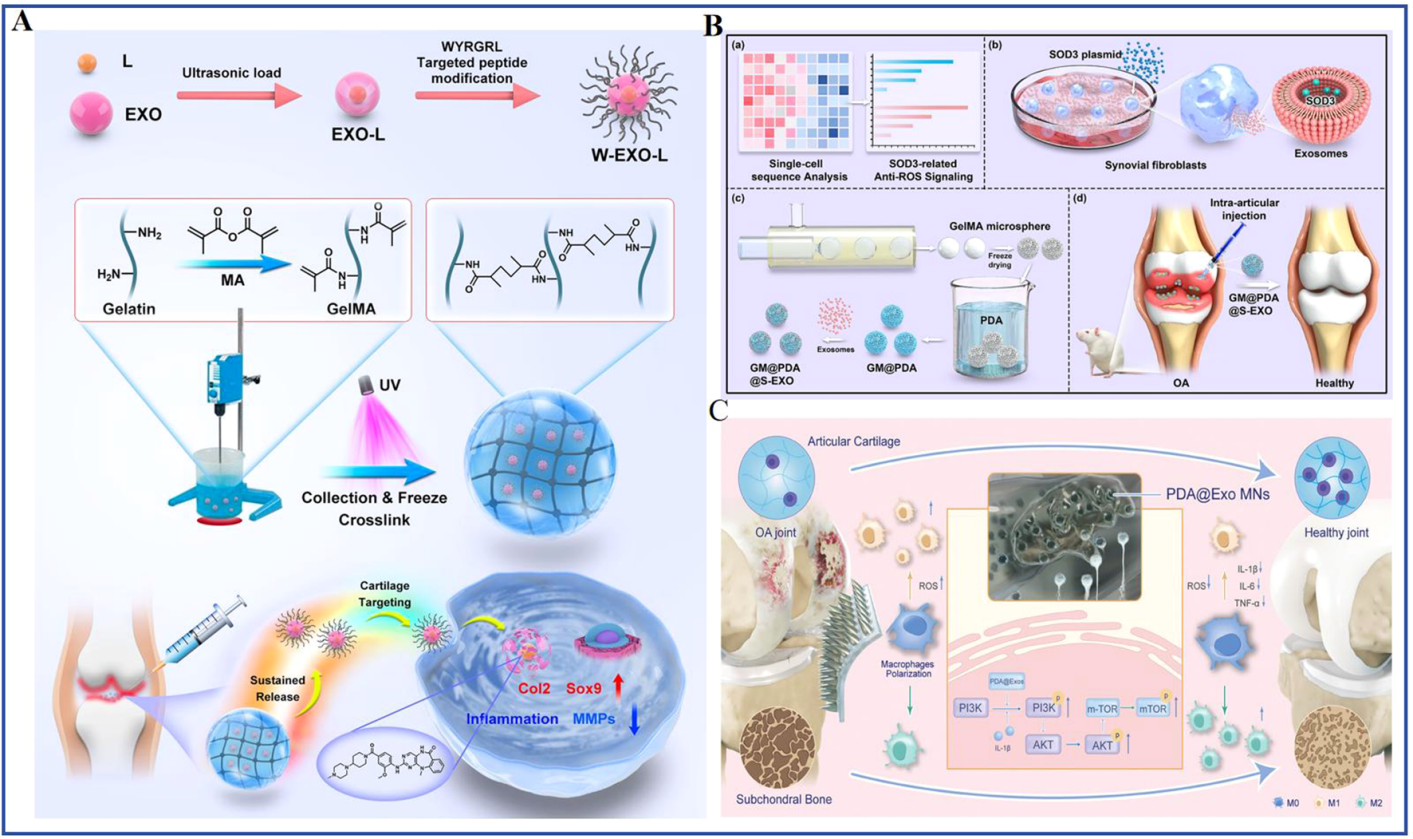
Engineered exosomes for knee osteoarthritis (KOA) repair. **(A)** Schematic illustration of an injectable, photocrosslinked spherical hydrogel encapsulating targeting peptide-modified engineered exosomes for the treatment of KOA. Adapted from Ref (57)., under a CC BY license. **(B)** Design of a hydrogel microsphere system loaded with SOD3-enriched exosomes for KOA therapy. **(a)** Analysis of single-cell sequencing data. **(b)** Preparation of SOD3-exosomes. **(c)** Fabrication of SOD3-exosome-loaded hydrogel microspheres. **(d)** Intra-articular administration of the prepared microspheres for KOA treatment. Adapted from Ref (60)., under a CC BY license. **(C)** Illustration of a microneedle-based delivery system containing polydopamine-coated exosomes for KOA therapy. Adapted from Ref (55). under a CC BY license.

Integrating exosomes with biomaterials overcomes rapid joint clearance and enables controlled release, targe. Hydrogel encapsulation is a primary strategy. MSC exosomes in GMOCS hydrogels restore ECM homeostasis and inhibit neutrophil extracellular traps via TGFβ1/Nrf2 activation ted delivery and additional microenvironment modulation (**Figure 4C**) (73). Magnetic polysaccharide-based microcarriers loaded with MSC exosomes rebalance ECM metabolism (downregulating MMP-13, upregulating collagen II and aggrecan) (111). Hydrogel microcarriers functionalized with a stem cell−recruiting peptide (SKPPGTSS) enable spatiotemporal exosome release and recruitment of endogenous BMSCs, achieving superior cartilage regeneration (**Figure 4D**) (74). More sophisticated systems include photocrosslinkable gelatin methacryloyl hydrogels for controlled release of LRRK2-IN-1-loaded, cartilage-targeted exosomes, synergistically delaying KOA progression (**Figure 6A**) (57); hydrogel microspheres embedding SOD3-loaded exosomes for sustained ROS neutralization (**Figure 6B**) (60); hyaluronic acid-based microneedle patches delivering polydopamine-incorporated exosomes to establish an anti-inflammatory microenvironment(**Figure 6C**) (55); and thermosensitive hydrogels (PLGA-PEG-PLGA) for Atf5 modRNA-loaded exosomes or M2 macrophage exosomes to promote autophagy and improve lymphatic drainage (83, 112).

Collectively, these biomaterial integration strategies address critical translational barriers-sustained retention, controlled release, targeted delivery and active microenvironment modulation-bringing engineered exosomes closer to clinical application for KOA.

## 6 Conclusions and prospects

Engineered exosomes, functionalized with therapeutic genetic materials, drugs, and targeting ligands, represent a promising frontier in the treatment of KOA. Advances in engineering strategies—including surface modification, genetic and physical cargo loading, and biomaterial encapsulation—have successfully addressed key limitations of native exosomes, such as low targeting specificity, limited payload capacity, poor stability, and short systemic circulation. These innovations have enhanced the ability of exosomes to precisely deliver therapeutics to affected joint tissues, modulate inflammatory responses, promote chondrocyte regeneration, restore ECM homeostasis, and even influence subchondral bone remodeling and pain signaling.

Despite their considerable therapeutic potential in preclinical studies, the clinical translation of engineered exosomes remains in its earliest stages. To date, only one randomized controlled trial (MR-13-24-017929) has published results evaluating exosome-based therapy specifically for KOA. Wang et al. conducted a randomized, double-blind, ascending dose study investigating hUC-MSC-exosomes in KOA patients. Their findings demonstrated that hUC-MSC-exosomes injection was safe, with no adverse consequences observed, and showed encouraging signs of efficacy based on clinical score improvements and MRI evaluations (113). This represents the first published clinical evidence supporting exosome therapy for KOA. Beyond this published trial, another study has explored the role of exosomes in the context of KOA treatment, though with important distinctions. For instance, a recent case-control study investigated whether the size of exosomes present in PRP was associated with clinical outcomes in KOA patients receiving PRP injections. The results suggested that PRP-derived exosome size may correlate with pain improvement at 12 weeks post-injection. While this study provides valuable mechanistic insights into PRP therapy, it does not constitute a trial of exosome-based therapeutics, as the intervention remained PRP rather than isolated exosomes (114).

Several registered interventional trials evaluating exosome-based therapies for KOA are listed on ClinicalTrials.gov (e.g., NCT05060107, NCT06431152, NCT06463132, NCT05261360). However, their results have not yet been published, and most remain in Phase 1 or Early Phase 1, primarily designed to establish safety and tolerability rather than disease-modifying efficacy. Some trials are listed with “Unknown status,” underscoring the challenges of early-stage clinical investigation in this emerging field. It is critically important to recognize that to date, no exosome-based therapy for KOA has demonstrated definitive structural or functional improvement in large-scale, multicenter randomized controlled trials. The field therefore awaits the outcomes of ongoing and future Phase II/III studies to establish clinical proof-of-concept, determine optimal dosing regimens, identify responsive patient populations, and assess long-term safety. This critical gap—where preclinical promise far exceeds clinical evidence—underscores the importance of the preclinical research reviewed herein, while also highlighting the urgent need for rigorous, well-designed clinical validation.

Beyond clinical trial status, the clinical translation of engineered exosomes faces several additional challenges that warrant in-depth consideration. First, the lack of standardized protocols for exosome isolation, purification, and characterization remains a fundamental barrier to clinical translation. Currently, the diversity of laboratory methods—including differential ultracentrifugation, size-exclusion chromatography, tangential flow filtration, and polymer-based precipitation—yields exosome populations with remarkable heterogeneity in terms of size, purity, yield, and functional properties. This methodological variability complicates cross-study comparisons and hinders efforts to establish reproducible therapeutic effects. Furthermore, characterization practices often rely on inconsistent parameters, with variable adherence to the Minimal Information for Studies of Extracellular Vesicles (MISEV) guidelines. Critical quality attributes such as particle-to-protein ratio, specific surface marker expression levels, and batch-to-batch consistency are frequently underreported. To address this, the field must urgently adopt harmonized standard operating procedures and establish well-defined release criteria for engineered exosomes intended for therapeutic use. Regulatory-grade characterization should include orthogonal methods for size distribution analysis (e.g., nanoparticle tracking analysis combined with tunable resistive pulse sensing), quantitative evaluation of surface marker profiles, assessment of cargo integrity and potency, and rigorous sterility and endotoxin testing. The development of reference materials and inter-laboratory validation studies will be essential to ensure reproducibility and comparability across different production sites and research groups.

Second, large-scale production and GMP manufacturing represent major hurdles. The transition from laboratory-scale isolation methods (e.g., ultracentrifugation) to industrial-scale production requires robust, reproducible, and cost-effective processes that comply with Good Manufacturing Practice (GMP) standards. Key challenges include maintaining batch-to-batch consistency, ensuring sterility, and establishing clearly defined Critical Quality Attributes (CQAs)—such as particle size distribution, surface marker expression, cargo integrity, and potency—for routine quality control. The development of scalable production platforms, such as tangential flow filtration or chromatography-based methods, is essential to meet the demands of future clinical trials and commercialization. Furthermore, the variability in laboratory methods—including exosome isolation, characterization, and engineering protocols—can contribute to remarkable differences in therapeutic properties, complicating efforts to establish standardized treatment protocols. The field urgently needs harmonized guidelines and consensus on standardized operating procedures, as advocated by the International Society for Extracellular Vesicles (ISEV).

Third, a comprehensive understanding of their pharmacokinetic (PK) and biodistribution profiles is imperative. Unlike small molecules or biologics, exosomes exhibit complex *in vivo* behavior influenced by their size, surface properties, and route of administration. Following intra-articular injection, the retention, clearance, and tissue penetration of exosomes within the joint microenvironment remain poorly characterized. Detailed investigations into their absorption, distribution, metabolism, and excretion (ADME) are needed to optimize dosing regimens and predict therapeutic outcomes. Advanced imaging modalities and labeling strategies may facilitate real-time tracking of exosome fate *in vivo*, providing critical insights into their pharmacokinetic behavior.

Fourth, immunogenicity and long-term safety must be rigorously evaluated. Although native exosomes are generally considered low-immunogenicity carriers, engineered modifications—such as surface functionalization with targeting ligands or heterologous cargo loading—may alter their immunogenic profile. Preclinical and clinical assessments should include comprehensive immunotoxicity studies to evaluate potential innate or adaptive immune responses, including cytokine release, complement activation, and anti-exosome antibody production. Furthermore, tumorigenicity risks, particularly for exosomes derived from proliferative cells or loaded with growth factors, require careful evaluation in appropriate animal models and long-term follow-up studies. These safety considerations are paramount for regulatory approval and clinical adoption.

Fifth, regulatory considerations pose a complex and evolving landscape. Engineered exosomes are typically classified as advanced therapy medicinal products (ATMPs) or biologic drugs, requiring adherence to stringent regulatory frameworks. Navigating these pathways demands close collaboration with regulatory agencies to define acceptable manufacturing controls, preclinical safety packages, and clinical trial designs. The absence of harmonized guidelines specific to exosome-based therapeutics further complicates development, underscoring the need for ongoing dialogue between researchers, manufacturers, and regulators. Establishing clearly defined CQAs and demonstrating consistent product quality will be essential for successful regulatory submissions.

Sixth, despite extensive investigations into the molecular content and functional effects of engineered exosomes, the precise mechanisms underlying their interaction with the pathological KOA microenvironment remain incompletely understood. Elucidating how exosomes modulate specific cell populations (e.g., chondrocytes, synoviocytes, macrophages) and signaling pathways (e.g., inflammatory, catabolic, or anabolic cascades) will inform rational design improvements and support mechanism-based potency assays for quality control.

Seventh, optimization of cargo loading and targeting efficiency remains an active area of research. Current engineering strategies—broadly categorized as pre-isolation modifications (e.g., genetic engineering of parent cells) and post-isolation modifications (e.g., chemical conjugation or physical loading)—each present distinct advantages and limitations. Pre-isolation methods enable stable cargo incorporation but may affect parent cell health or exosome yield; post-isolation methods offer greater control over cargo type and quantity but may compromise exosome integrity or targeting fidelity. Developing hybrid or next-generation approaches that maximize both loading capacity and functional performance is a key priority for the field.

Eighth, the majority of preclinical efficacy studies have been conducted in small animal models (e.g., rodents) or *in vitro* systems, with limited evaluation in large animal models (e.g., rabbits, dogs, or sheep) that more closely recapitulate human joint anatomy, biomechanics, and disease progression. Bridging this gap is essential to better predict clinical efficacy and inform trial design. Future studies should prioritize large animal validation before advancing to human trials.

Finally, optimal dosing, delivery strategies, and treatment regimens for clinical application remain undefined. Variables such as exosome dose, injection frequency, formulation (e.g., suspension vs. hydrogel encapsulation), and combination with existing therapies (e.g., analgesics or physical therapy) require systematic investigation in well-controlled trials. Addressing these knowledge gaps will be critical for translating preclinical promise into clinical practice.

Collectively, the cumulative advancements in the engineered exosomes hold promise of creating precision-targeted therapeutic agents capable of navigating the complex biological environment of the KOA. By leveraging these sophisticated methodologies, there is a realizable prospect of developing a new class of engineered exosomes interventions that can address the intricate challenges posed by KOA pathologies. However, with only one published randomized controlled trial to date, the field must now focus on rigorous, stepwise clinical validation—including the requisite Phase II/III trials—to translate this promise into a proven clinical reality.
