# Supplementary material for: Unlocking the potential of engineered exosomes for knee osteoarthritis therapy
Source: Front Immunol. 2026 Apr 29;17:1820504. doi: 10.3389/fimmu.2026.1820504 (PMC13167554; doi:10.3389/fimmu.2026.1820504)
Supplement: Supplementary file 1 [file Table1.docx]

| Study | Animal model | Sample size | Exosome Source | Engineering Strategy | Exosome Yield | Targeting  Efficiency | Frequency of treatment | Dosage | Delivery route | Therapeutic Outcomes (In vivo) |
| --- | --- | --- | --- | --- | --- | --- | --- | --- | --- | --- |
| Inflammation |  |  |  |  |  |  |  |  |  |  |
| Wang et al.[1] | Rat OA model induced by the combination of ACLT and MMT | n=20 per group | FLSs | Exosomes from miR-146a transfected with FLSs | Not reported | Not reported | Twice a week for 4–8 weeks after KOA modeling | 100 μg/rat, | Intra-articular injections | Chondrocytes apoptosis↓；  Cartilage degeneration↓;  M2 macrophages↑;  M1 macrophages↓;  Proinflammatory cytokines↓;  TLR4/TRAF6/NF-κB pathway↓;  OARSI Score: Not reported |
| Zhou et al.[2] | Rat OA model induced by the combination of ACLT and MMx | n=5 per group | SFCs | Exosomes from miR-126-3p transfected with SFCs | Not reported | Not reported | Once per week for ten weeks | 40 μl of 500 μg/ml | Intra-articular injections | Cartilage degeneration↓;  Chondrocytes apoptosis↓；  Inflammation↓;  OARSI Score: Not reported |
| Lai et al. [3] | Rat OA model induced by the combination of ACLT and MMx | n=6 per group | SFBs | Exosomes from miR-214-3p transfected with SFBs | Not reported | Not reported | Weekly (post-op week 4-10) | 40 μL/injection | Intra-articular injections | Cartilage degeneration↓;  Subchondral bone structure↑;  Proinflammatory cytokines↓;  OARSI Score: Not reported |
| Sang et al. [4] | DMM-induced rat OA model | n=10 per group | Primary chondrocytes | Encapsulation of naïve exosomes within thermosensitive F127-HA hydrogel for sustained release | Exosome suspension concentration: 8.14 × 10¹³ particles/mL | Not reported | Once every 2 weeks, starting from 4 weeks post-surgery, for a total of 3 injections | 200 μL per injection | Intra-articular injections | Cartilage degeneration↓;  Cartilage matrix synthesis↑;  Synovial inflammation↓;  M2 macrophages↑;  M1 macrophages↓;  Proinflammatory cytokines↓;  Anti-inflammatory cytokines↑;  OARSI Score ↓ |
| Wan et al. [5] | DMM-induced mouse OA model | n=10 per group | BMSCs | LRRK2-IN-1 loading + WYRGRL surface modification + GelMA encapsulation | Not reported | Enhanced chondrocyte uptake; Prolonged joint retention to 14 days (IVIS) | Once every 2 weeks, starting from day 3 post-surgery, for a total of 8 weeks (4 injections) | 10 μL per injection | Intra-articular injection | Cartilage degeneration↓;  Anabolism↑;   Catabolism↓;  Subchondral bone degeneration↓;  OARSI Score↓ |
| Zhang et al. [6] | MIA-induced OA rat model | n=6 per group | BMSCs | Puerarin loaded into BMSC-Exo via co-incubation + ultrasonication | Not reported | Time-dependent cellular uptake | Twice weekly (Day 1 and 4 each week) for 4 weeks (total 8 injections) | 50 μL per injection | Intra-articular injection | Cartilage degeneration↓;  Subchondral bone degeneration↓;  Proinflammatory cytokines↓;  NF-κB pathway↓;  OARSI Score↓ |
| Cao et al. [7] | DMM-induced OA model in mice | n=3 per group | Synovial fibroblasts | SOD3 mimic transfection + GM@PDA encapsulation | Not reported | PDA modification ↑ loading capacity (13.6 vs. 3.9 μg/mg) & prolonged release (14 vs. 3 days); GM@PDA@S-EXO ↑ cartilage SOD3 expression vs. free S-EXO (+78%) | Once every two weeks, starting one-week post-surgery, for 8 weeks (total 4 injections) | 10 μL per injection | Intra-articular injection | Cartilage degeneration↓;  SOD3 delivery↑;  ACAN expression↑; |
| Li et al. [8] | ACLT-induced OA rat model | Not reported | MSCs | PDA@Exo composite + HAMA microneedle fabrication | Not reported | Penetration: ~159 μm (skin)/800 μm (exosomes); sustained release: 58.85% at 12 h, over 7 days | Starting 4 weeks post-surgery, every 3 days for 4 weeks (total ~9-10 applications) | Topical MN application (dose not specified; in vitro up to 400 μg/mL) | Transdermal microneedle patch application | Cartilage degeneration↓;  Subchondral bone degeneration↓;  OARSI Score↓ |
| Wu et al.[9] | DMM-induced OA model in mice | n=8 per group | IPFP-MSCs | IPFP-MSCs treated with TNF-α (75 ng/mL) for 48 h prior to exosome collection | TNF-α ↑ exosome secretion ~1.8-fold (NTA) via PI3K/AKT/ATG16L1 pathway | Not reported | Once every other week, starting 4 weeks post-surgery, for 4 weeks (total 2 injections) | 10 μL per injection containing 5 × 10¹⁰ particles/mL | Intra-articular injection | Synovial inflammation↓;  Cartilage degeneration↓;  Gait parameters↑;  Osteophyte formation↓;  OARSI Score: Not reported |
| Chen et al.[10] | ACLT-induced OA rat model | n=12 per group | HucMSCs | IL-1β priming (10 ng/mL, 24 h) + HAMA hydrogel microsphere encapsulation | ↑ vs. naïve Exos (p < 0.001); fold-change not specified | Cellular uptake confirmed; sustained release ≥15 days; | Weeks 5 and 6 post-surgery (2 injections total) | Not reported | Intra-articular injection | Cartilage degeneration↓;  Subchondral bone degeneration↓;  OARSI Score↓ |
| Wang et al.[11] | DMM-induced rat OA model | n=6 per group | BMSCs | TGF-β1 preconditioning | Not reported | Not reported | A single intra-articular injection. | 1*10^10^exosome particles per injection | Intra-articular injection. | Systemic inflammation↓;  Chondrocyte number↑；  Cartilage degeneration↓;   MAPK6 expression↑；  OARSI Score: Not reported |
| Cell function and ECM homeostasis |  |  |  |  |  |  |  |  |  |  |
| Liu et al. [12] | Rat OA model induced by the combination of ACLT and DMM | n=10 per group | hUSCs | Exosomes from miR-140-5p transfected with hUSCs | Exosome concentration: 5.1 × 10¹⁰ particles/mL (NTA); diameter: 135.5 nm (average), 97.2% within 30-150 nm range; protein concentration not specified | Chondrocyte uptake confirmed; ↑ miR-140 in recipient cells; VEGFA validated as direct target; ↓ VEGFA protein & tube formation | Once weekly for 4 or 8 weeks, starting at 4 weeks post-surgery (total 4 or 8 injections) | 100 μL per injection containing 10¹¹ particles/mL (total 1 × 10¹⁰ particles per injection) | Intra-articular injection. | Chondrocyte number↑;  Cartilage degeneration↓;  VEGFA expression↓;  ECM synthesis ↑; |
| Wang et al. [13] | DMM-induced rat OA model | n=3 per group | ADSCs | Exosomes from miR-140-5p transfected with ADSCs | Not reported | Rapid chondrocyte uptake within 6 h, fluorescence persisted for 7 days (in vitro); joint retention ≥7 days in normal/DMM mice, | Weekly intra-articular injections for 10 weeks | 20 μL per injection | Intra-articular injection | Cartilage degeneration↓;   Apoptosis ↓;  ECM remodeling↑;   OARSI score↓ |
| Wang et al, [14] | Cold water stimulation-induced mouse OA model | n=5 per group | SMSCs | Exosomes from miR-155-5p transfected with SMSCs | Exosome concentration: 10¹¹ particles/mL  Size: 100–120 nm | Cellular uptake confirmed | Daily intra-articular injections for 2 weeks, starting 20 days after OA induction | 30 μL per injection containing 10¹¹ particles/mL (total 3 × 10⁹ particles per injection) | Intra-articular injection | Apoptosis ↓;  Chondrocyte number↑;  OARSI score ↓；  Cartilage degeneration↓; |
| Mao et al. [15] | Collagenase VII-induced mouse OA model | n = 10 per group | MSCs | Exosomes from miR-92a-3p transfected with MSCs | Exosome size: 50–150 nm  Exosomes concentration: 500 μg/mL | Cellular uptake confirmed | Days 7, 14, and 21 post-OA induction (3 injections total) | 15 μL per injection containing 500 μg/mL exosomes (total 7.5 μg exosomal protein per injection) | Intra-articular injection | ECM degradation↓;  ECM synthesis↑;  Cartilage degeneration↓;  OARSI Score: Not reported |
| Tao et al. [16] | Rat OA model induced by the combination of ACLT and DMM | n = 10 per group | SMSCs | Exosomes from miR-140-5p transfected with SMSCs | Size: 30–150 nm (TEM/NTA); Markers: CD9⁺/CD63⁺/CD81⁺/Alix⁺; Stock: 1 mg/mL; miRNA loading: fluorescence-confirmed (ex/em 532/580 nm), efficiency not specified | Exosomes were internalized by chondrocytes and localized in the perinuclear region | Weekly injections from week 5 to week 8 post-surgery (4 injections total) | 100 μL per injection containing 10¹¹ particles/mL (total 10¹⁰ particles per injection) | Intra-articular injection | Chondrocyte number↑;  ECM degradation↓;  ECM synthesis↑;  Cartilage degeneration↓;   OARSI score↓ |
| Liang et al. [17] | DMM-induced rat OA model | n = 5 per group | Dendritic cells | CAP surface display + miR-140 electroporation | Not reported | Chondrocyte uptake↑; Joint retention >24 h; penetration to deep cartilage | Once weekly for 4 weeks, starting at 4 weeks post-surgery | 100 μL per injection containing 100 μg exosomal protein (1 mg/mL stock) | Intra-articular injection | ECM degradation↓;  ECM synthesis↑;  Chondrocyte number↑;  OARSI score↓ |
| Zhang et al. [18] | ACLT-induced OA mouse model | n = 6 per group | Expi293F cells | CAP lipid insertion + siMMP13 electroporation | Size: 100–109 nm; Zeta: -7.8 to -10.1 mV; Markers: CD9⁺/Hsp70⁺/TSG101⁺; CAP coupling: 58.3% (~9600 peptides/exosome); siRNA loading: 34.96% (~4113 molecules/exosome); cup-shaped (TEM | Chondrocyte uptake↑; Joint retention 93.3%;  Deep cartilage penetration; | Once weekly for 4 weeks, starting at 4 weeks post-surgery | 100 μL per injection | Intra-articular injection | Cartilage degeneration↓;  Subchondral bone degeneration↓;  M2 macrophages↑;  M1 macrophages↓;  OARSI score↓  ECM degradation↓; |
| Xu et al.[19] | DMM-induced rat OA model | n = 5 per group | Bone marrow-derived dendritic cells | E7-Lamp2b engineering + KGN electroporation | Size: 100–300 nm; CD63⁺/CD81⁺; KGN loading: 40% (electroporation); ~50% release at 24 h; stable ≥7 days | Selective SF-MSC uptake; even cytosolic distribution; in vivo migration to deep cartilage by day 7 with sustained KGN retention | Weekly intra-articular injections for 4 weeks, starting at 4 weeks post-surgery (rats sacrificed at week 5) | 100 μL per injection | Intra-articular injection | Cartilage degeneration↓;  ECM degradation↓;  OARSI score↓ |
| Zhao et al.[20] | Rat OA model induced by the combination of ACLT and DMM;  ACLT-induced OA mouse model | Rats: n = 6 per group;  Mice: n = 5 per group | Subcutaneous fat-derived MSCs | CAP-Lamp2b engineering + miR-199a-3p electroporation | Native Exosome:1.6×10¹⁰ particles/mL, CD9⁺/CD81⁺/Alix⁺/Tsg101⁺/Calnexin⁻, cup-shaped. CAP-Exos${}^{SC}$: 90–170 nm, CD9⁺/Alix⁺/Tsg101⁺/Lamp2b⁺/Calnexin⁻, cup-shaped. miR-199a-3p loading: ~40%. | Chondrocyte uptake↑;  Preferential delivery to chondrocytes over synovial cells;  Deep cartilage penetration in vivo | Rats (first study): Once weekly for 6 weeks, starting 3 weeks post-surgery  Rats (second study): Antagomir: twice weekly for 3 weeks (weeks 2–4);  Mice: weekly  for 6 weeks (weeks 5–10)  • Mice: Once weekly for 4 weeks, starting 6 weeks post | Rats: 50 μL per injection containing 2 × 10¹⁰ particles/mL (total 1 × 10⁹ particles per injection)  • Mice: 10 μL per injection containing 1 × 10¹⁰ particles/mL (total 1 × 10⁸ particles per injection);  Antagomir: 50 μL per injection | Intra-articular injection | Cartilage degeneration↓;  OARSI score↓;  Autophagy↑;  mTOR protein↑ |
| Luo et al. [21] | DMM-induced rat OA model | n = 6 per group | BMSCs | Fucoidan preconditioning | Protein: 5.30±0.29 (MSCs-Exo) vs 5.82±0.47 μg/10⁶ cells (F-MSCs-Exo) (ns); Size: 156.7 vs 144.0 nm; Cup-shaped; CD9⁺/CD63⁺/CD81⁺/TSG101⁺/Calnexin⁻; Cellular uptake confirmed (PKH67) | Not reported | Weekly intra-articular injections for 4 weeks, starting at 4 weeks post-surgery | 10 μL per injection | Intra-articular injection | MMP-13↓;  Cartilage degeneration↓;  OARSI score↓ |
| Ma et al.[22] | DMM-induced rat OA model | n = 5 per group | BMSCs | ATF5-modRNA transfection + PLGA-PEG-PLGA encapsulation | ~1 μg/μL; 30–200 nm; TSG101⁺/CD9⁺/CD63⁺; ATF5 mRNA enriched | ~90% uptake in 3 h; joint retention 15–21 days; sustained release 15 days; >12-fold ATF5 protein increase | Every 2 weeks after surgery (injections at weeks 0, 2, 4, 6, 8, 10; total 6 injections over 12 weeks) | 100 μL per injection containing 10 particles/μL exosomes (total 10³ particles per injection) | Intra-articular injection | ATF5 and ClpP protein ↑;  Cartilage degeneration↓;  ECM degradation↓;  ECM synthesis↑;  OARSI score↓  Subchondral bone degeneration↓; |
| Liu et al.[23] | Rat OA model induced by the combination of ACLT and DMM; | n = 5 per group | hUSCs | Exosomes from miR-92a-3p transfected with hUSCs | 124.8 ± 52.4 nm; CD63⁺/CD9⁺/Calnexin⁻; cellular uptake confirmed | miR-140 in recipient cells↑; joint retention >10 days; cartilage penetration confirmed | Weekly intra-articular injections for 4 weeks, starting at week 5 post-surgery | 100 μL per injection | Intra-articular injection | Gait parameters↑;  Subchondral bone degeneration↓;  Cartilage degeneration↓;  ECM degradation↓;  ECM synthesis↑;   Mitophagy↑;  OARSI score↓ |
| Shi et al.[24] | DMM-induced OA mouse model | n = 5 per group | AMSCs | Exosomes from tsRNA-12391  transfected with AMSCs | Spherical, 50–200 nm; ALIX⁺/TSG101⁺; | Cellular uptake confirmed;  Co-localization with tsRNA-12391-FAM;  Uptake efficiency not quantified | Once weekly for 4 weeks, starting at 4 weeks post-surgery | 10 μL per injection containing 1 μg/μL exosomal protein (total 10 μg protein per injection) | Intra-articular injection | Mitophagy↑;  Cartilage degeneration↓;  OARSI Score: Not reported |
| Shao et al.[25] | Collagenase-2-induced OA rat model | n = 6 per group | BMSCs | PTH (1-34) preconditioning | 4.26–5.15×10¹¹ particles/mL; 60–150 nm; cup-shaped; CD9⁺/CD63⁺/TSG101⁺/CD90⁺/CD14⁻; uptake confirmed | Not reported | Weekly intra-articular injections for 4 weeks | 10 μg/mL | Intra-articular injection | OARSI score↓  Cartilage degeneration↓;  ECM degradation↓;  ECM synthesis↑; |
| Meng et al.[26] | ACLT-induced OA rat model | n = 5 per group | ADSCs | Tropoelastin preconditioning | 1.81–2.68×10¹⁰ particles/mL; TE ↑ yield 2.5× & 1.7×; 50–200 nm; TSG101⁺/CD81⁺/Calnexin⁻; ↑ uptake vs. Exo (p < 0.001) | Not reported | Single intra-articular injection at 4 weeks post-surgery; rats sacrificed at 8 weeks post-surgery | 10 μL of exosome solution containing 1 × 10¹⁰ particles/mL | Intra-articular injection | Cartilage degeneration↓;  ECM degradation↓;  ECM synthesis↑;  Inflammatory infiltration↓;  OARSI score↓ |
| Zhang et al.[27] | DMM-induced OA mouse model | n = 5 per group | BMSCs | DECM preconditioning | Size: ~120 nm; Conc: 8.67×10¹⁰/mL (dECM) vs 7.27×10¹⁰/mL (control); cup-shaped; HSP70⁺/CD63⁺/TSG101⁺ | Uptake confirmed in vitro/vivo; dECM-exosomes↑ miR-3473b in chondrocytes | Twice weekly for 4 weeks, starting at 4 weeks post-surgery (total 8 injections) | 10 μL per injection containing 10¹⁰ particles/mL exosomes |  | Cartilage degeneration↓;  ECM degradation↓;  ECM synthesis↑;  PTEN expression ↓;  OARSI score↓ |
| Qiu et al. [28] | Surgically induced OA mouse model (specific surgical method not detailed in the provided text) | Not reported | BMSCs | Curcumin preconditioning | TEM: round, 50–150 nm; CD9⁺/CD63⁺/CD81⁺; yield measured (BCA) but not specified | Not reported | Not reported | Not reported | Intra-articular injection | Apoptosis ↓;  NF-κB and ROCK1 protein ↓;  Cartilage degeneration↓;  OARSI Score: Not reported |
| Chang et al. [29] | ACLT-induced OA rat model | n = 7–8 per group | ADSCs | Hypoxic microenvironment preconditioning | Size (NTA): ~130 nm; Spherical/rotund shape;  Positive for CD9, CD63, CD81, ALIX, TSG101; Negative for α-tubulin | Uptake confirmed in vitro/vivo; | Weekly intra-articular injections for 7 consecutive weeks (starting 2 weeks post-ACLT surgery) | 10⁷–10⁹ particles/mL | Intra-articular injection | Cartilage degeneration↓;  Synovial inflammation↓；  ECM degradation↓;  ECM synthesis↑; |
| Li et al. [30] | Rat OA model induced by the combination of ACLT and DMM; | n = 5 per group | M2 macrophages | Hypoxic microenvironment preconditioning | TEM: cup-shaped; Markers: CD9⁺/CD63⁺/TSG101⁺; NTA: ~140–160 nm; Hypoxia ↑ exosome production and ↑ chondrocyte uptake | Uptake confirmed in vitro/vivo; | Once weekly for 4 weeks, starting at 4 weeks post-surgery (total 4 injections). | 100 μL intra-articular injection containing 1 × 10⁹ particles/mL exosomes | Intra-articular injection | Synovial inflammation↓；  Cartilage degeneration↓;  ECM degradation↓;  ECM synthesis↑;  OARSI score↓   Apoptosis ↓;  Proliferation ↑;  Migration ↑; |
| Zhou et al. | Rat OA model induced by the combination of ACLT +DMM+MCL; | Not reported | BMSCs | GMOCS hydrogel encapsulation | TEM: cup/sphere-shaped, 50–200 nm; CD9⁺/CD63⁺/CD81⁺/TSG101⁺; sustained release over 14 days | Uptake by neutrophils confirmed; sustained release in vivo | Single injection | 15 μL | Intra-articular injection | Apoptosis ↓; Proliferation ↑; Migration ↑;  ECM degradation↓;  ECM synthesis↑;  NETs formation ↓;  ROS ↓;  Nrf2 pathway ↑; Cartilage degeneration ↓; OARSI score ↓; Osteophytes ↓; |
| Yang et al. [31] | Surgically induced OA rat model (surgical method not detailed in the provided text) | Not reported | UC-MSCs | Magnetic polysaccharide microcarriers (HAMA/CSMA) + Fe₃O₄@MgSiO₃ nanoparticles for exosomes capture + diclofenac sodium (DS) loading | Porous microcarriers; Exo captured by Fe₃O₄@MgSiO₃ nanoparticles; sustained release over 14 days | Exosomes retention in joint cavity: up to 14 days; uptake by chondrocytes confirmed | Intra-articular injections at 2-week intervals | 20 μL | Intra-articular injection | Cell viability ↑;  Apoptosis ↓;  ECM degradation↓;  ECM synthesis↑;   OARSI score ↓  Cartilage degeneration↓; |
| Song et al. [32] | ACLT-induced OA mouse model | n = 6 per group | M2 macrophages | Thermosensitive hydrogel encapsulation: | TEM: disc-shaped, 30–150 nm; TSG101⁺/CD81⁺; Calnexin⁻; sustained release up to 12 days | M2Exo internalized by LECs in vitro (Mem Dye-Green labeling); sustained release in vivo via hydrogel | Biweekly intra-articular injections (total 2 injections) | 0.1 mg exosomes loaded into HP hydrogel | Intra-articular injection | Cartilage degeneration ↓;  OARSI score ↓;  Synovial lymphatic vessels ↑  Lymphangiogenesis ↑;  LEC viability ↑; LEC proliferation ↑; LEC apoptosis ↓; LEC migration ↑ |
| Subchondral Bone Remodeling |  |  |  |  |  |  |  |  |  |  |
| Li et al. [33] | DMM-induced OA mouse model | n = 6 per group | Chondrocytes | Cyclic tensile strain stimulation of chondrocytes to secrete exosomal miR-9-5p; miR-9-5p overexpression via transfection | TEM: sphere-shaped bilayer membrane, 50–150 nm (mean 92.84 nm); CD63⁺/CD81⁺/HSP70⁺; yield measured but not specified | DiO-labeled exosomes internalized by osteoblasts in vitro (confocal microscopy) | Once weekly for 10 weeks (starting 1-week post-DMM surgery) | 5 μL | Intra-articular injection | Cartilage degeneration ↓; Subchondral bone remodeling ↓;  ECM degradation↓;  ECM synthesis↑;  Cartilage lesion depth ↓  OARSI score ↓; |
| Wang et al. [34] | ACLT-induced OA mouse model | n = 6 per group | BMSCs | TGF-β1  preconditioning | TEM: typical morphology (Fig. S1); CD63⁺/CD9⁺ (Western blot); NTA: size distribution characterized; concentration: 1×10¹¹ particles/mL; yield measured but not specified | Exosomes localized to subchondral bone | Daily intra-articular injections for 8 weeks | 10 μL (1×10¹¹ exosome particles/mL) per injection | Intra-articular injection | Cartilage degeneration ↓; Calcified cartilage thickness ↓; Proteoglycan loss ↓; Subchondral bone remodeling ↓; Osteoclastogenesis ↓; MAPK pathway↓;  Smad2/3 phosphorylation ↓;  H-type vessels ↓;  PDGF-BB ↓; |
| Pain pathway |  |  |  |  |  |  |  |  |  |  |
| Lu et al. [35] | DMM-induced OA mouse model | n = 5 per group | MSCs | Exosome mimetics (EMs) generated by serial extrusion of hMSCs; miR-204 loaded via electroporation | TEM: ~100 nm; CD9⁺/CD81⁺/TSG101⁺; average size increased ~8% after miR-204 loading; loading efficiency >80%; release kinetics: miR-204 levels in chondrocytes peaked then decreased to baseline by 72h | EMs taken up by chondrocytes in vitro (IF, flow cytometry); in vivo retention in knee joint for at least 5 days (IVIS) | Every two weeks for 8 weeks (total 4 injections) | 15 μg EMs^(miR-204) per injection | Intra-articular injection | Pain behavior ↓;  Nerve ingrowth ↓;  Cartilage degeneration ↓;  OARSI score ↓;  ECM degradation↓;  ECM synthesis↑; |
| Liebmann et al. [36] | MIA-induced OA mouse model | n = 8 per group | IFP-MSCs | Genetic modification: IFP-MSCs transduced with AAV vector containing GFP-labeled CGRP antagonist (CGRP₈₋₃₇) gene, followed by FACS sorting to generate aCGRP IFP-MSCs | TEM: characterized but images not shown; NTA: 3-4×10⁹ particles/mL; CD9⁺/CD81⁺/TSG101⁺ (flow cytometry); size distribution characterized; LncRNA cargo analysis identified 24 unique LncRNAs | Not reported | Single intra-articular injection (4 days post-MIA) | 100 μL (3-4×10⁹ EVs/mL) per injection | Intra-articular injection | Pain behavior ↓;  Cartilage degeneration ↓;  Collagen organization ↑;  ECM degradation ↓;  OARSI Score: Not reported |
| Wu et al. [37] | MIA-induced OA rat model | n = 3 per group | HEK293T cells | Co-transfection of VSV-G-ZsGreen1 and HAS2-mCherry plasmids into HEK293T cells; | TEM: cup-like, ~100 nm; DLS: 30-200 nm; zeta potential: -22.6 mV; VSV-G and HAS2 colocalized on exosome surface (IF); yield measured by BCA but not specified | pH-responsive membrane fusion via VSV-G at pH 6.0 (avoiding lysosomal degradation); V-H Exo delivered HAS2 to chondrocyte membranes (colocalization with DIO); fluorescence intensity significantly higher than HAS2 Exo (flow cytometry) | 1-week post-MIA for OA model | 100 μg exosomes in 50-100 μL PBS per injection | Intra-articular injection | Pain threshold ↑;  Inflammatory factors ↓ ; Synovial thickness ↓;  M2 macrophages↑;  M1 macrophages↓;  Cartilage degeneration ↓;  OARSI Score: Not reported |

Table 1. Production Characteristics and Targeting Efficiency of Engineered Exosomes for KOA Therap
