# Supplementary material for: Unlocking the potential of engineered exosomes for knee osteoarthritis therapy
Source: Front Immunol. 2026 Apr 29;17:1820504. doi: 10.3389/fimmu.2026.1820504 (PMC13167554; doi:10.3389/fimmu.2026.1820504)
Supplement: Supplementary file 2 [file Table2.docx]

**Table 2. Quantitative Comparison of Engineered Exosome Studies in KOA Pathological Domains**

| Pathological Domain | Number of studies | Key Mechanistic Pathways | Representative Molecular Targets |
| --- | --- | --- | --- |
| Inflammation | 12 | TLR4/TRAF6/NF-κB signaling inhibition; M1→M2 macrophage polarization; ROS neutralization; PI3K/Akt/mTOR pathway activation; IκBα/IKKα/β/p65 phosphorylation inhibition; MAPK6 suppression | miR-146a, miR-126-3p, miR-214-3p, miR-147b, miR-766-3p, miR-205-5p, miR-135b, LRRK2-IN-1, puerarin, SOD3, polydopamine (PDA), TNF-α, IL-1β, IL-6, IL-17, TLR4, TRAF6, NF-κB, MAPK6, IκBα, IKKα/β, p65 |
| ECM Homeostasis | 30 | miRNA-mediated regulation (miR-140-5p/VEGFA, miR-212-5p/ELF3, miR-155-5p/Runx2, miR-338-3p/Runx2, miR-486-5p/Runx2, miR-92a-3p/Wnt/β-catenin, miR-127-3p/Wnt/β-catenin, miR-95-3p/HDAC2/8); Surface engineering for targeted delivery (CAP-Lamp2b, CPC, E7-Lamp2b); Autophagy induction (miR-199a-3p/mTOR, miR-146-5p/TRAF6/PI3K/AKT/mTOR, Atf5/mTOR/Ulk1); Mitophagy activation (miR-140/CAPN1/Parkin, tsRNA-12391/ATAD3A/PINK1); Cellular preconditioning (PTH/miR-3473b, PTH/let-7a-5p, tropoelastin/miR-451-5p, dECM/miR-3773b/PTEN/AKT, TGF-β1/miR-135b/Sp1, curcumin/miR-143/miR-124/ROCK1/TLR9/NF-κB, cinnamaldehyde/NF-κB/MAPK, hypoxia/miR-381-3p/122-5p/143-3p/206/30a-5p/22-3p/151a-5p, hypoxia/M2 macrophages/miR-124-3p/STAT3); Biomaterial synergy (GMOCS hydrogel/TGFβ1/Nrf2, magnetic microcarriers, M2 macrophage exosomes/hydrogel/lymphatic drainage, SKPPGTSS peptide/endogenous BMSC recruitment) | VEGFA, ELF3, Runx2, Wnt/β-catenin, HDAC2/8, MMP-13, ADAMTS-5, COL2A1, aggrecan, CAP-Lamp2b, CPC, E7-Lamp2b, siRNA against MMP13, IL-1RA, kartogenin (KGN), mTOR, Ulk1, TRAF6, PI3K, AKT, Atf5, CAPN1, Parkin, ATAD3A, PINK1, PTH, let-7a-5p, miR-451-5p, PTEN, Sp1, ROCK1, TLR9, STAT3, TGFβ1, Nrf2 |
| Subchondral Bone | 3 | osteoclast/osteoblast differentiation, H-type vessel formation | PDGF-BB, KLF5, miR-9-5p, RANK, OSCAR, miR-1227-5p |
| Pain Modulation | 3 | Neuro-cartilage crosstalk, neuropeptide signaling (SP, CGRP), joint lubrication | SP, CGRP, LRP1, Sp1, CD10, TNF, TLR4, MAPK8 |

This quantitative comparison reveals that while subchondral bone remodeling and pain modulation are critically important in KOA pathophysiology—with subchondral bone abnormalities contributing to approximately 40% of biomechanical failure and pain dominating the clinical burden—the current literature on engineered exosomes targeting these domains remains substantially less developed than research on inflammation and ECM homeostasis. This evidence gap identifies important opportunities for future investigation.
